# Entropy and Entransy Dissipation Analysis of a Basic Organic Rankine Cycles (ORCs) to Recover Low-Grade Waste Heat Using Mixture Working Fluids

**DOI:** 10.3390/e20110818

**Published:** 2018-10-24

**Authors:** Yong-qiang Feng, Qian-hao Luo, Qian Wang, Shuang Wang, Zhi-xia He, Wei Zhang, Xin Wang, Qing-song An

**Affiliations:** 1 School of Energy and Power Engineering, Jiangsu University, Zhenjiang 212013, China; 2 Key Laboratory of Efficient Utilization of Low and Medium Grade Energy, Tianjin University, Ministry of Education of China, Tianjin 300072, China

**Keywords:** organic Rankine cycles (ORCs), entropy, entransy dissipation, mixture working fluids

## Abstract

Mixture working fluids can reduce effectively energy loss at heat sources and heat sinks, and therefore enhance the organic Rankine cycle (ORC) performance. The entropy and entransy dissipation analyses of a basic ORC system to recover low-grade waste heat using three mixture working fluids (R245fa/R227ea, R245fa/R152a and R245fa/pentane) have been investigated in this study. The basic ORC includes four components: an expander, a condenser, a pump and an evaporator. The heat source temperature is 120 °C while the condenser temperature is 20 °C. The effects of four operating parameters (evaporator outlet temperature, condenser temperature, pinch point temperature difference, degree of superheat), as well as the mass fraction, on entransy dissipation and entropy generation were examined. Results demonstrated that the entransy dissipation is insensitive to the mass fraction of R245fa. The entropy generation distributions at the evaporator for R245/pentane, R245fa/R152a and R245fa/R227ea are in ranges of 66–74%, 68–80% and 66–75%, respectively, with the corresponding entropy generation at the condenser ranges of 13–21%, 4–17% and 11–21%, respectively, while those at the expander for R245/pentane, R245fa/R152a and R245fa/R227ea are approaching 13%, 15% and 14%, respectively. The optimal mass fraction of R245fa for the minimum entropy generation is 0.6 using R245fa/R152a.

## 1. Introduction

With the increasing shortage of fossil energy and the escalating demand for energy, extensive attention has been paid to the renewable energy technologies. Several methods applicable to utilize renewable sources [1,2,3,4,5] have been studied, such as the organic Rankine cycle (ORC), Kalina cycle (using ammonia water) and trilateral cycle. Among them, ORC [6,7,8,9,10,11,12,13] is a promising method to convert low/medium-grade thermal energy into power. A large number of academic researches have been carried out, such as the choice of working fluids, the application of different configurations of ORC and economic analysis. In recent years, entropy generation has gradually played an important role in the research of ORC. Groniewsky et al. [14] used the Redlich–Kwong equation of state to predict the temperature-entropy saturation boundary of working fluids. They found that a limiting isochoric heat capacity may exist between dry and wet fluids. Li et al. [15] conducted an entropy generation analysis on the ORC system. Zhu et al. [16] applied the entropy analysis on the ORC system and found that for R123 and N-pentane, the optimal evaporation temperature and optimized output power are different.

In addition, some researchers showed their interest in mixture working fluids, because mixture working fluids have a temperature glide at a two-phase zone, resulting in a better match with heat sources and reducing the system irreversible loss. Heberle et al. [17] conducted the second-law efficiency of ORC system using isobutane/isopentane and R227ea/R245fa. Zhang et al. [18] used R245fa and 0.7isopentane/0.3R245fa to contrast the thermodynamic performance of a regenerative ORC. Feng et al. [19] compared the ORC’s performance using R245fa, pentane and R245fa/pentane. They found that mixture working fluids show a lower thermodynamic performance and a moderate economic performance than the pure working fluids. Dong et al. [20] investigated the thermal efficiency of high-temperature ORC system using zeotropic working fluids. Xiao et al. [21] conducted a multi-objective optimization on the ORC system with a multi-objective function by the incorporation of four single-objective functions. They found that the performance of mixture working fluids was not always better than that of pure working fluids, which is similar to the results obtained by Feng et al. [22]. Lecompte et al. [23] investigated the performance of ORC with zeotropic mixtures based on a second-law analysis. They found that the evaporator accounts for the highest energy loss. Chys et al. [24] obtained the optimal mass fraction of zeotropic mixtures for an ORC system. Angelino et al**.** [25] analyzed an ORC system using zeotropic mixtures and indicated that selecting the optimum composition of mixture working fluids is a method to design an efficient ORC system. Shu et al. [26] conducted the thermal efficiency and exergy loss analysis of ORC system using three pure hydrocarbons and two retardants, stating that zeotropic mixtures presented better thermodynamic performance than the pure working fluids. Oyewunmi et al. [27] assessed large-glide fluorocarbon working-fluid mixtures by the statistical associating fluid theory for potentials of variable range (SAFT-VR) in ORC, and they found that SAFT-VR Mie produced relatively higher accurate thermodynamic properties of working fluids. Liu et al. [28] investigated the effect of condensation temperature glide on ORC performance using zeotropic mixtures. Garg et al. [29] studied the performance of ORC using isopentane, R245fa and their mixtures, and they found that the maximum system efficiency of 10–13% can be obtained at an optimal expansion ratio of 7–10. Oyewunmi et al. [30] studied the thermodynamic performance of ORC system using the zeotropic working fluid. They found that the mixture working fluids do not have a better thermodynamic performance than the pure working fluids. Yang et al. [31] examined the effect of zeotropic mixtures for ORC system to recover a diesel engine exhaust energy, indicating that R402B yielded the maximum net power output of 24.65 kW.

Recently, a few researchers applied the entransy analysis on the ORC system. Cheng et al. [32] discussed the entransy expressions of the three thermodynamic cycles. Entransy is a quantity to describe the “potential energy of heat” during the process of heat exchanging, which has been widely used to optimize the heat conversion system. Li et al. [33] established an integrated optimization method based on the entransy theory, and they found that the optimal operating parameters can be determined with the optimal pinch point temperature difference (PPTD) of 5 °C. Li et al. [34] investigated the entransy dissipation/loss-based optimization of a two-stage ORC using R245fa. They found that the two-stage ORC could increase the average evaporating temperature, and thus decline the entransy dissipation rate. 

As mentioned above, few researchers fulfilled the work on the entransy and entropy analyses on the ORC system using mixture working fluids. Accordingly, the purposes of this study are: (a) examining the effects of operating parameters on the entropy generation and entransy dissipation; and (b) investigating the effects of mass fraction on the entropy generation and entransy dissipation. 

## 2. Analysis of the ORC System

**Figure 1:**
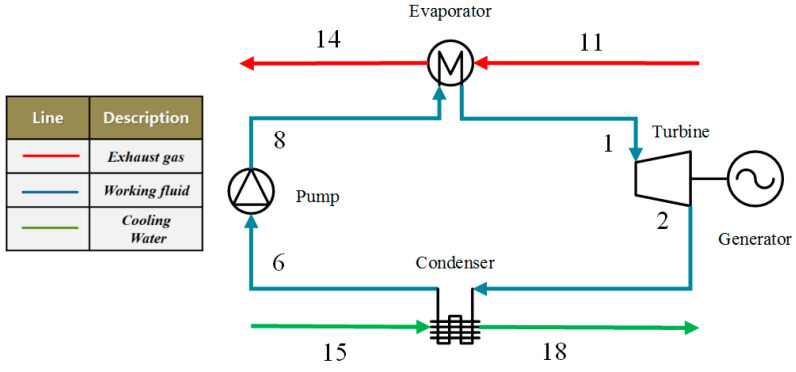
Schematic diagram of the basic organic Rankine cycle (ORC) system to recover low-grade waste heat.

**Figure 2:**
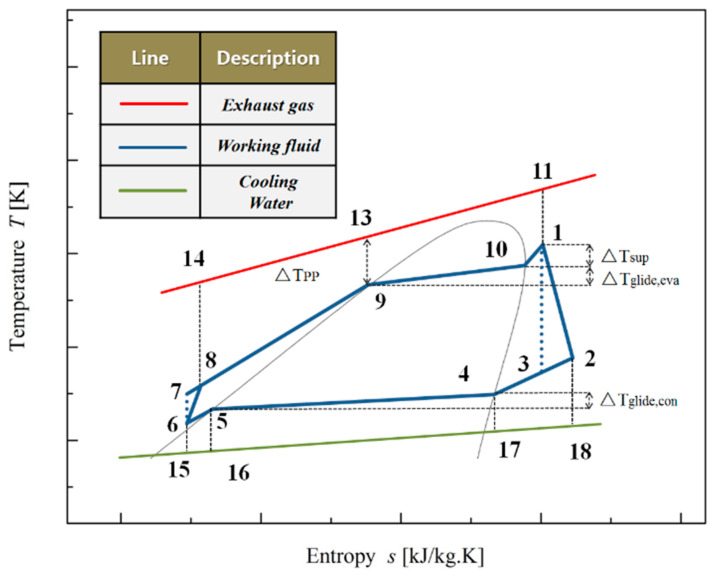
Temperature–entropy *(T-s)* diagram for the basic ORC system.

The schematic diagram of the basic ORC system to recover low-grade waste heat is shown in Figure 1, which includes an evaporator, an expander, a condenser, and a working fluid pump [35]. The thermodynamic process for the ORC system using mixture working fluids (showing the so-called dry characteristic) is illustrated on the temperature–entropy (*T–s*) diagram, as shown in Figure 2. It should be noted that the connecting points at a blue curve of 10, 9, 5 and 4 are the saturation points. The whole system is stable without leakages and heat losses. ORC is set to recover the waste heat, which is represented by a red line in Figure 2. Additionally, the working fluid and cooling water are represented by a blue line and a green line, respectively. A temperature of 120 °C and a mass flow rate of 0.33 kg/s are used for the simulated heat source, while the cooling water is used to condensate the working fluids with a condensate temperature of 20 °C.

In the evaporator, the working fluid is heated and vaporized by waste heat, and then the high pressure vapor (state 1) flows into the expander and its enthalpy is converted into work. The low-pressure vapor (state 2) exits the expander and is led to the condenser where it is liquefied by cooling water. Similarly, the liquid working fluid is available at the condenser outlet (state 6), and then it is pumped back to the evaporator, and a new cycle begins.

Compared to the pure working fluids, the main advantage of mixtures as ORC working fluids stems from their non-isothermal phase transitions during vaporization and condensation, and hence effectively matches the heat source and cooling water. The corresponding *T–s* diagram for the ORC system using mixture working fluids is shown in Figure 2.

The pump power (${\dot{W}}_{p}$) can be expressed as:

(1) $${\dot{W}}_{p}={\dot{m}}_{\mathrm{wf}}\left(h_{8}-h_{6}\right)/\eta_{p}\;$$

where $\eta_{p}$ is the mechanical efficiency of pump.

Through the *T–s* plot, the ORC evaporator heat transfer rate (${\dot{Q}}_{\mathrm{eva}}$) and the condenser heat transfer rate (${\dot{Q}}_{\mathrm{con}}$) can be given as follows:

(2) $${\dot{Q}}_{\mathrm{eva}}={\dot{m}}_{\mathrm{wf}}(h_{1}-h_{8})\;$$

(3) $${\dot{Q}}_{\mathrm{con}}={\dot{m}}_{\mathrm{wf}}(h_{2}-h_{6})\;$$

where ${\dot{m}}_{wf}$ is the mass flow rate of mixture working fluids, which can be defined as:

(4) $$m_{\mathrm{wf}}=m_{h}\left(h_{11}-h_{14}\right)/\left(h_{1}-h_{8}\right)\;$$

Since mixture working fluids have non-isothermal phase transitions in vaporization and condensation, the temperature glide of evaporator and condenser (as shown in Figure 2) can be expressed as:

(5) $$\Delta T_{\mathrm{glide},\mathrm{eva}}=T_{10}-T_{9}\;$$

(6) $$\Delta T_{\mathrm{glide},\mathrm{con}}=T_{4}-T_{5}\;$$

The temperature difference between the evaporator outlet temperature and saturation temperature corresponding to evaporator outlet pressure is superheat ($\Delta T_{\sup}$), which can be given as:

(7) $$\Delta T_{\sup}=T_{1}-T_{10}\;$$

The PPTD in the ORC evaporator ($\Delta T_{\mathrm{PP}}$) can be defined as:

(8) $$\Delta T_{\mathrm{PP}}=T_{13}-T_{9}\;$$

## 3. Modeling

### 3.1. Entropy Modeling

Based on previous research [12,19,22], the following equation of the entropy generation rate (${\dot{S}}_{g}$) can be obtained.

The entropy generation of evaporator and condenser can be expressed:

(9) $${\dot{S}}_{g,\mathrm{eva}}={\dot{m}}_{h}(s_{14}-s_{11})+{\dot{m}}_{\mathrm{wf}}(s_{1}-s_{8})\;$$

(10) $${\dot{S}}_{g,\mathrm{con}}={\dot{m}}_{\mathrm{con}}(s_{18}-s_{15})+{\dot{m}}_{\mathrm{wf}}(s_{6}-s_{2})\;$$

In addition, entropy generation of expander and pump can be defined as:

(11) $${\dot{S}}_{g,\exp}={\dot{m}}_{\mathrm{wf}}(s_{2}-s_{3})\;$$

(12) $${\dot{S}}_{g,p}={\dot{m}}_{\mathrm{wf}}(s_{8}-s_{6})\;$$

Based on Equations (14)–(16), the entropy generation of system can be given as:

(13) $${\dot{S}}_{g,\mathrm{sys}}={\dot{S}}_{g,\mathrm{eva}}+{\dot{S}}_{g,\mathrm{con}}+{\dot{S}}_{g,\exp}+{\dot{S}}_{g,p}\;$$

### 3.2. Entransy Modeling

Based on the researches by Cheng and Liang [36], the entransy loss rate can be given as follows:

(14) $${\dot{G}}_{\mathrm{loss}}={\dot{G}}_{H}-{\dot{G}}_{L}\;$$

where ${\dot{G}}_{H}$ is the entransy flows into the system, ${\dot{G}}_{L}$ is the entransy flows out of the system, and they can be defined in ORC system as:

(15) $${\dot{G}}_{H}=\frac{1}{2}C_{h}(T_{h,\mathrm{in}}^{2}-T_{0}^{2})\;$$

(16) $${\dot{G}}_{L}=\frac{1}{2}C_{c}(T_{0}^{2}-T_{c,\mathrm{in}}^{2})+{\dot{Q}}_{0}T_{0}\;$$

Combining Equations (19) and (20), the Equation (18) can be changed into:

(17) $${\dot{G}}_{\mathrm{loss}}=\frac{1}{2}C_{h}(T_{h,\mathrm{in}}^{2}-T_{0}^{2})+\frac{1}{2}C_{c}(T_{c,\mathrm{in}}^{2}-T_{0}^{2})-{\dot{Q}}_{0}T_{0}\;$$

(18) $${\dot{G}}_{\mathrm{loss}}=\frac{1}{2}C_{h}(T_{h,\mathrm{in}}^{2}-T_{0}^{2})+\frac{1}{2}C_{c}(T_{c,\mathrm{in}}^{2}-T_{0}^{2})+\dot{W}T_{0}\;$$

The total entransy dissipation rate of the ORC system includes three parts. The first one is the entransy dissipation rate resulting from heat transfer between the hot stream and the working fluid:

(19) $${\dot{G}}_{\mathrm{diss},\mathrm{eva}}=\frac{1}{2}C_{h}(T_{h,\mathrm{in}}^{2}-T_{h,\mathrm{out}}^{2})-\left[\frac{1}{2}Q_{8,9}(T_{8}^{}+T_{9}^{})+\frac{1}{2}Q_{9,10}(T_{9}+T_{10})+\frac{1}{2}Q_{1,8}(T_{1}+T_{8})\right]\;$$

As the same principle goes, the second brace is the entransy dissipation rate due to heat transfer between the working fluid and the cold stream:

(20) $${\dot{G}}_{\mathrm{diss},\mathrm{con}}=\frac{1}{2}Q_{2,4}(T_{2}+T_{4})+\frac{1}{2}Q_{4,5}(T_{4}+T_{5})+\frac{1}{2}Q_{5,6}(T_{5}+T_{6})-\frac{1}{2}C_{h}(T_{c,\mathrm{out}}^{2}-T_{c,\mathrm{in}}^{2})\;$$

The last part is the entransy dissipation rate due to dumping the used streams into the environment.

(21) $${\dot{G}}_{\mathrm{diss},\mathrm{env}}=\left[\frac{1}{2}C_{h}(T_{h,\mathrm{out}}^{2}-T_{0}^{2})+\frac{1}{2}C_{c}(T_{c,\mathrm{out}}^{2}-T_{0}^{2})\right]-\left[C_{h}(T_{h,\mathrm{out}}^{}-T_{0}^{})T_{0}+C_{c}(T_{c,\mathrm{out}}-T_{0})T_{0}\right]\;$$

Combining Equations (23)–(25), we can get:

(22) $${\dot{G}}_{\mathrm{diss},\mathrm{sys}}=\left\{\frac{1}{2}C_{h}(T_{h,\mathrm{in}}^{2}-T_{h,\mathrm{out}}^{2})-\left[\frac{1}{2}Q_{8,9}(T_{8}^{}+T_{9}^{})+\frac{1}{2}Q_{9,10}(T_{9}+T_{10})+\frac{1}{2}Q_{1,8}(T_{1}+T_{8})\right]\right\}\;-\left\{\frac{1}{2}C_{h}(T_{c,\mathrm{out}}^{2}-T_{c,\mathrm{in}}^{2})-\left[\frac{1}{2}Q_{2,4}(T_{2}^{}+T_{4}^{})+\frac{1}{2}Q_{4,5}(T_{4}+T_{5})+\frac{1}{2}Q_{5,6}(T_{5}+T_{6})\right]\right\}\;+\left\{\left[\frac{1}{2}C_{h}(T_{h,\mathrm{out}}^{2}-T_{0}^{2})+\frac{1}{2}C_{c}(T_{c,\mathrm{out}}^{2}-T_{0}^{2})\right]-\left[C_{h}(T_{h,\mathrm{out}}^{}-T_{0}^{})T_{0}+C_{c}(T_{c,\mathrm{out}}-T_{0})T_{0}\right]\right\}\;$$

## 4. Global Model

### 4.1. Working Fluid Selection

The selection of working fluid is crucial because working fluids have a great influence on the safety operation condition, economic efficiency and environmental impact. The primary fluid selection criteria for the ORC included: a high decomposition temperature to withstand the high-temperature exhaust gas and high boiling point under atmospheric pressure to easily release condensing heat to cooling water. Meantime, environmental protection, safety and economic characteristics still need to be taken into account. Considering the above criteria, proper working fluids were primarily selected and the basic thermodynamic properties of the fluids are listed in Table 1. Meanwhile, Feng et al. [22] investigated the thermoeconomic performance of mixture working fluids using R245fa/R227ea and R245fa/pentane. Wang et al. [37] presented an analysis of low-temperature solar ORC using R245fa/R152a. Therefore, three mixture working fluids, R245fa/R227ea, R245fa/R152a and R245fa/pentane, were selected in this study.

### 4.2. Assumptions

In order to simplify the computing simulation study appropriately, general assumptions [38,39] should be used in this study:

(a) The system is in a steady state.
(b) Heat and friction losses, as well as the potential and kinetic energy, are neglected.
(c) There are no pressure drops in the heat exchangers, condensers and pipes.
(d) The ambient condition is set to 0.1 MPa.
(e) The temperature of cooling water is set to 283.15 K.
(f) The isentropic efficiencies of the expander and the pump are both set to be 0.8.

The main assumptions for the ORC system are listed in Table 2.

## 5. Results and Discussion

To better understand the effects of system parameters on entransy dissipation and entropy generation, three mixture working fluids (0.5R245fa/0.5R227ea, 0.5R245fa/0.5R152a and 0.5R245fa/0.5pentane) were chosen in Section 5.1 and Section 5.2. It should be noted that the thermodynamic properties of mixture working fluids were obtained from the NIST Refprop [40]. According to the *T–s* plot, the operation parameters (PPTD, degree of superheat, evaporator outlet temperature and condenser temperature) have a significant influence on the system performance. Therefore, the effects of operation parameters on entransy dissipation and entropy generation were addressed in Section 5.1 and Section 5.2, respectively. The effects of mass fraction on entransy dissipation and entropy generation were examined in Section 5.3.

### 5.1. Effects of Operation Parameters on Entransy Dissipation

**Figure 3:**
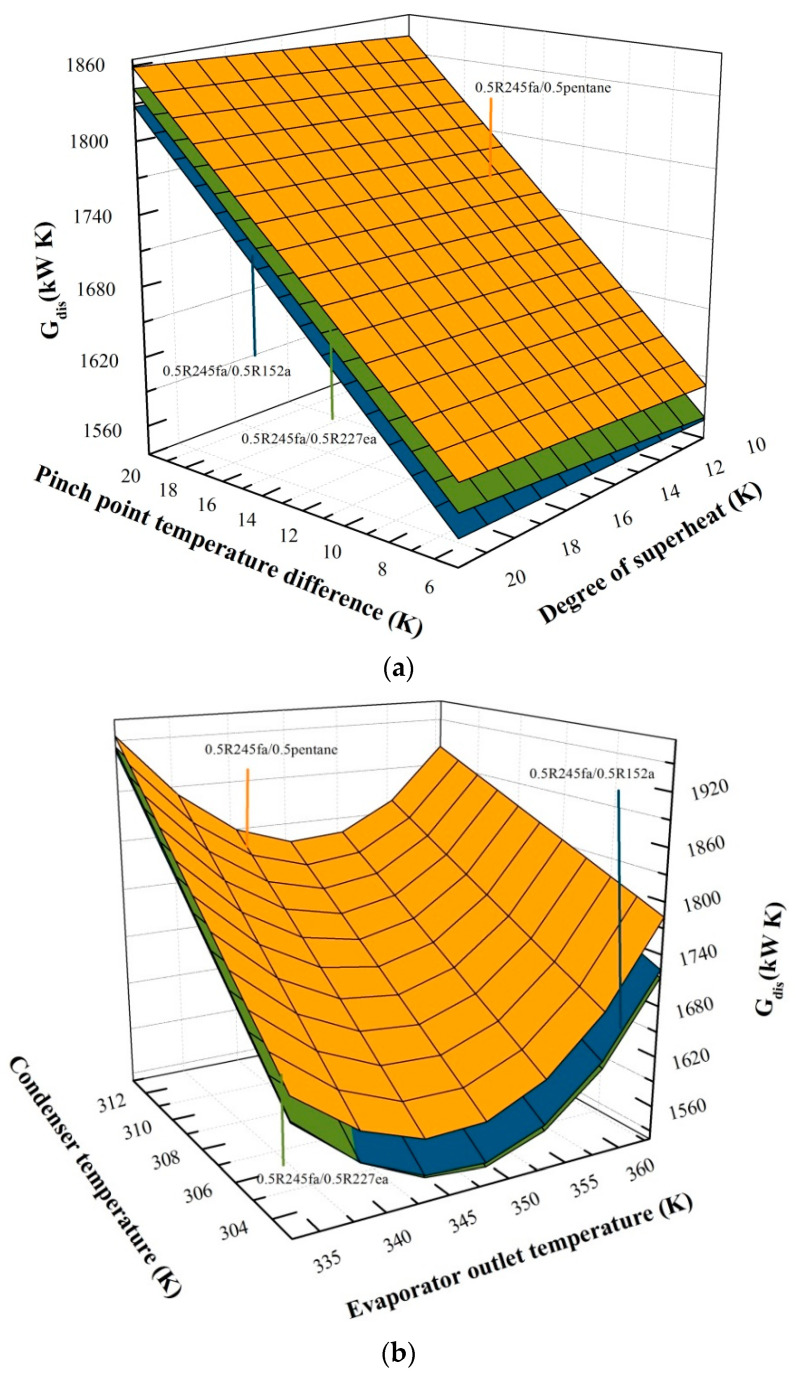
(**a**) Variation of entransy dissipation with pinch point temperature difference and the degree of superheat. (**b**) Variation of entransy dissipation with the evaporator outlet temperature and condenser temperature.

The variations of entransy dissipation with PPTD and the degree of superheat using 0.5R245fa/0.5R227ea, 0.5R245fa/0.5R152a and 0.5R245fa/0.5pentane are displayed in Figure 3a. The evaporator outlet temperature and condenser temperature are set to be 60 and 30 °C, respectively. The PPTD varies from 5 °C to 20 °C, and the degree of superheat is in a range of 10–20 °C.

Obviously, the entransy dissipation is mainly affected by the PPTD. As the pinch point temperature increases, the entransy dissipation linearly increases because of the increasing of irreversible loss between the heat source and working fluid. At the same time, entransy dissipation is hardly affected by the changes in degree of superheat. As noted in Equation (20), when the PPTD increases, the temperature points ($T_{2}$, $T_{4}$, $T_{5}$,$T_{6}$, $T_{8}^{}$, $T_{9}^{}$, $T_{10}$ and $T_{1}$) are unchanged, whereas the heat source outlet temperature deceases, resulting in the decline in the working fluid mass flow rate, and causing eventually the increase in entransy dissipation. Therefore, entransy dissipation presents an increasing trend with the PPTD. Moreover, it is worth mentioning that 0.5R245fa/0.5R227ea has a better performance than 0.5R245fa/0.5R152a. 

Figure 3b illustrates the effects of evaporator outlet temperature and condenser temperature on the entransy dissipation using 0.5R245fa/0.5R227ea, 0.5R245fa/0.5R152a and 0.5R245fa/0.5pentane. The PPTD and degree of superheat are set to be 5 °C and 10 °C, respectively. The evaporator outlet temperature varies from 60 °C to 90 °C and the condenser temperature varies from 30 °C to 40 °C. 

The entransy dissipation for three mixture working fluids owns a similar behavior of an increase with the condenser temperature but has a nonlinear variation with the evaporator outlet temperature. As expressed in Equation (17), when the evaporator outlet temperature increases, the temperature points ($T_{8}^{}$, $T_{9}^{}$, $T_{10}$ and $T_{1}$) increase, whereas the working fluid mass flow rate declines, ensuring the decline in the heat transfer rate, and eventually causing the decrease in entransy dissipation at first. However, as the evaporator outlet temperature continues to rise, the decline of the mass flow rate gradually occupies a dominant position compared to the rise of temperature, and thus the entransy dissipation increases gradually. The evaporator outlet temperature corresponding to the lowest entransy is in a range of 70–80 °C. 

It can also be found that the entransy dissipation keeps decreasing with the condenser temperature. The reason is that the temperature difference declines between the working fluid and cooling water, resulting in the decrease in the irreversible loss. In Equation (18), as the condenser temperature increases, the temperature points ($T_{2}$, $T_{4}$, $T_{5}$ and $T_{6}$) increase, while the mass flow rate has no change. Accordingly, the entransy dissipation represents a rising trend with the condenser temperature. Moreover, 0.5R245fa/0.5pentane yields the highest *G*_dis_, followed by 0.5R245fa/0.5R152a. In addition, 0.5R245fa/0.5R227ea owns the lowest G_dis_ with the evaporator outlet temperature of 80 °C and the condenser temperature of 30 °C.

### 5.2. Effects of Operation Parameters for Entropy Generation

**Figure 4:**
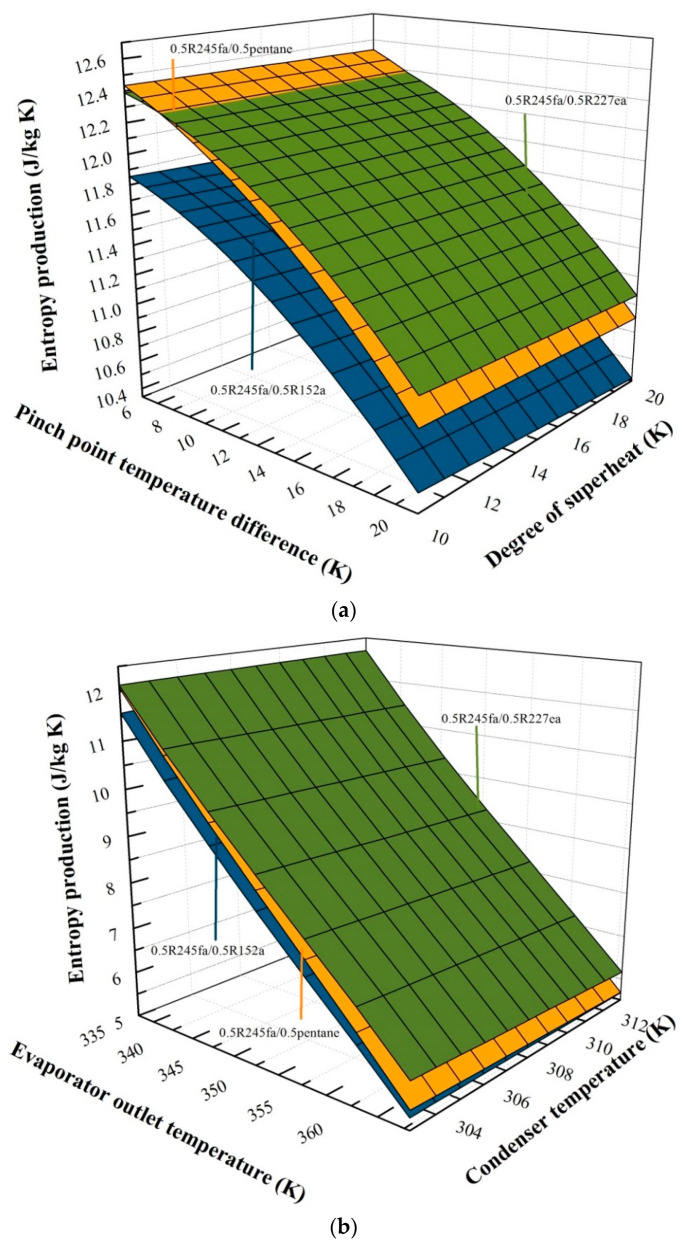
(**a**) Variation of entropy generation with the pinch point temperature difference and the degree of superheat. (**b**) Variation of entropy generation with the evaporator outlet temperature and condenser temperature.

Figure 4a reveals the variation of entropy generation with the PPTD and degree of superheat using 0.5R245fa/0.5R227ea, 0.5R245fa/0.5R152a and 0.5R245fa/0.5pentane. The evaporator outlet temperature is 60 °C and the condenser temperature is 30 °C. The PPTD varies from 5 °C to 20 °C, and the degree of superheat varies from 10 °C to 20 °C. The entropy generation for the three working fluids has a similar behavior to the degree of superheat and PPTD. At first, with the increase of PPTD, 0.5R245fa/0.5pentane has the highest entropy generation. However, as the point temperature difference further increases, 0.5R245fa/0.5pentane gradually highlights its advantages. 0.5R245fa/0.5R227ea presents the highest entropy generation, followed by 0.5R245fa/0.5pentane, and 0.5R245fa/0.5R152a has the lowest entropy generation. In Equation (4), the mass flow rate of heat source keeps constant and $h_{14}$ decreases with the increase of the PPTD, and therefore the mass flow rate of working fluids declines. As expressed in Equations (9)–(13), when the PPTD increases, the mass flow of working fluids decreases, and therefore the generated entropy at the evaporator, condenser, expander and pump decline.

The variation of entropy generation with the evaporator outlet temperature and condenser temperature are illustrated in Figure 4b. The PPTD is 5 °C and the degree of superheat is 10 °C. The evaporator outlet temperature varies from 60 °C to 90 °C, and the condenser temperature varies from 30 °C to 40 °C. The increase in the evaporator outlet temperature results in a decrease in entropy generation. However, with the condenser temperature increasing, entropy generation decreases slightly. As stated in Equation (4), when evaporator outlet temperature increases, the heat source mass flow rate has no change and the value of $h_{14}$ keeps rising, resulting in the decline in the mass flow rate. In Equations (9)–(12), the entropy generation of system declines with the working fluid mass flow rate. Furthermore, it can be found that 0.5R245fa/0.5R227ea presents the highest entropy generation, followed by 0.5R245fa/0.5pentane and 0.5R245fa/0.5R152a. For example, for a specified evaporator temperature of 65 °C and a condenser temperature of 30 °C, the entropy generation is 10.991 J/kg K for 0.5R245fa/0.5R227ea, 10.865 J/kg K for 0.5R245fa/0.5pentane, and 10.420 J/kg K for 0.5R245fa/0.5R152a.

### 5.3. Effects of Mass Fraction on Entransy Dissipation and Entropy Generation

**Figure 5:**
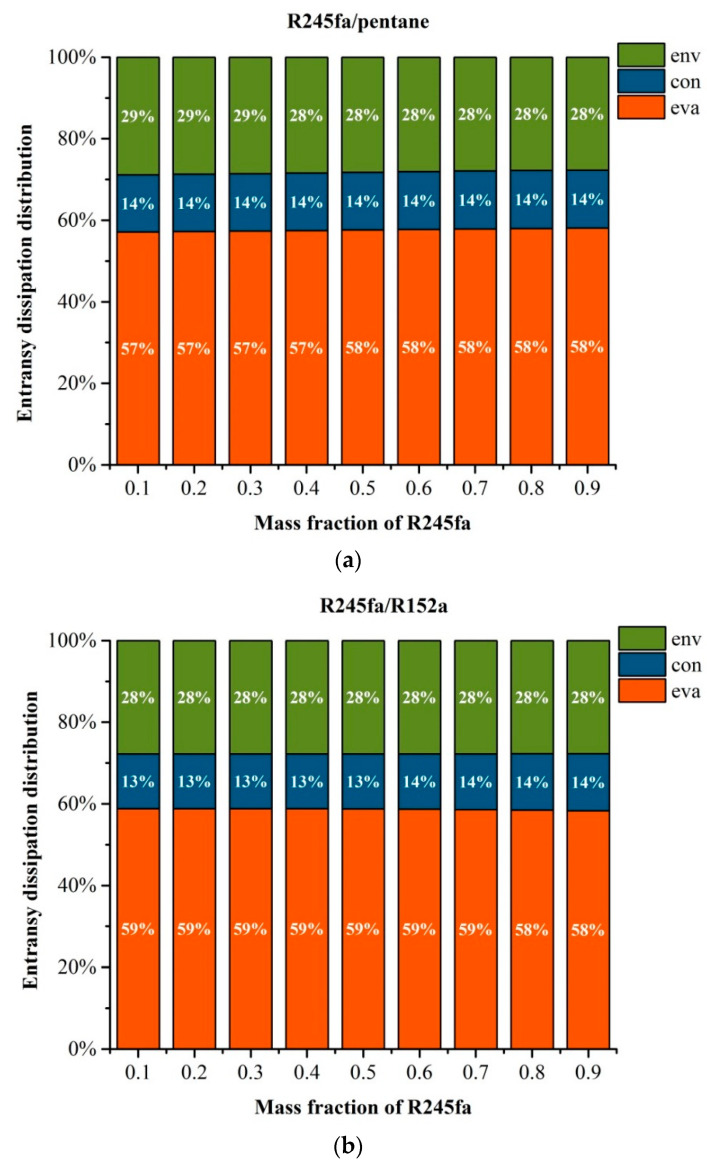

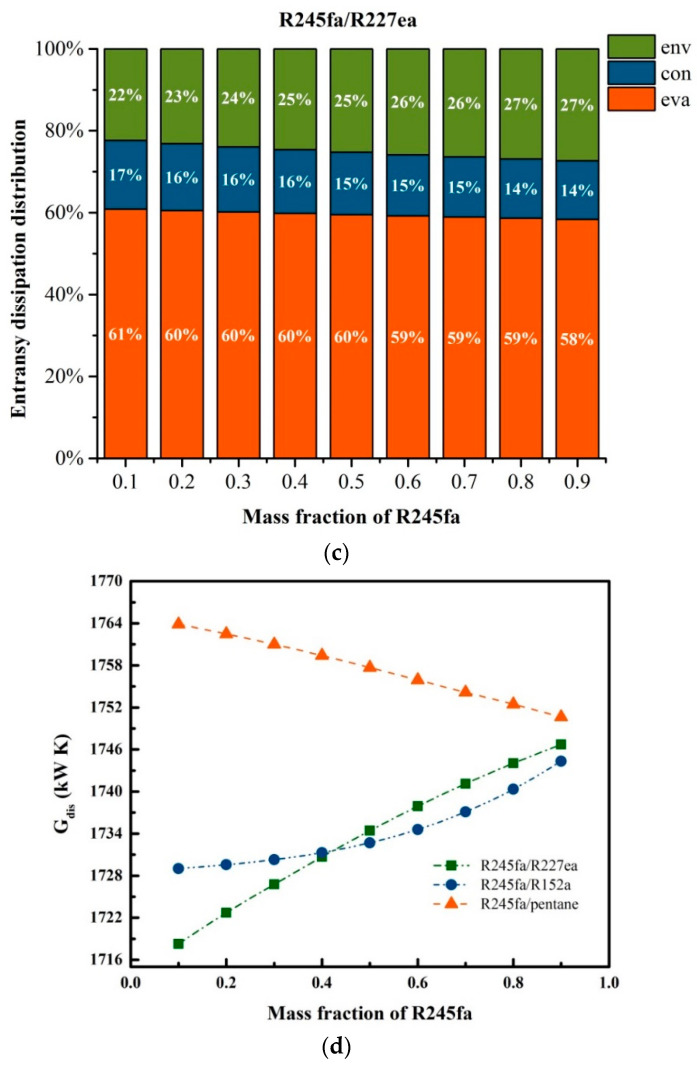
Variation of entransy dissipation with the mass fraction of R245fa: (**a**) R245fa/pentane; (**b**) R245fa/R152a; (**c**) R245fa/R227ea. And (**d**) variation of entransy dissipation with the mass fraction of R245fa using R245fa/pentane, R245fa/R152a and R245fa/R227ea.

The variation of entransy dissipation for three working fluids with the mass fraction of R245fa is displayed in Figure 5a–c. The PPTD, degree of superheat, evaporator outlet temperature and condenser temperature are set to be 5, 10, 60 and 30 °C, respectively. 

As noted in Equations (21)–(24), the entransy dissipation of system is affected mainly by G_diss,env_, G_diss,con_ and G_diss,eva_. Figure 5a–c reveals that the evaporator owns the largest proportion of entransy dissipation in the ORC system. It can also be found that the entransy dissipation for the three mixture working fluids have a similar trend. As for R245fa/pentane, the proportions of evaporator, condenser and environment are 58%, 14%, and 28%, respectively, which are approaching the values for R245fa/R152a. Meanwhile, the proportions of evaporator, condenser and environment for R245fa/R227ea are 60%, 15%, and 15%, respectively, indicating that the entransy dissipation is insensitive to the working fluid type. Moreover, the entransy dissipation almost has no change with the mass fraction of R245fa, representing that the mass fraction of mixture working fluids has a small impact on the entransy dissipation. Figure 5d shows the trend of the entransy dissipation of the three mixture working fluids with the variation of the mass fraction of R245fa. Obviously, the entransy dissipations of the R245fa/R227ea and R245fa/R152a keep rising, whereas that of R245fa/pentane yields a reverse trend with the mass fraction of R245fa. 

**Figure 6:**
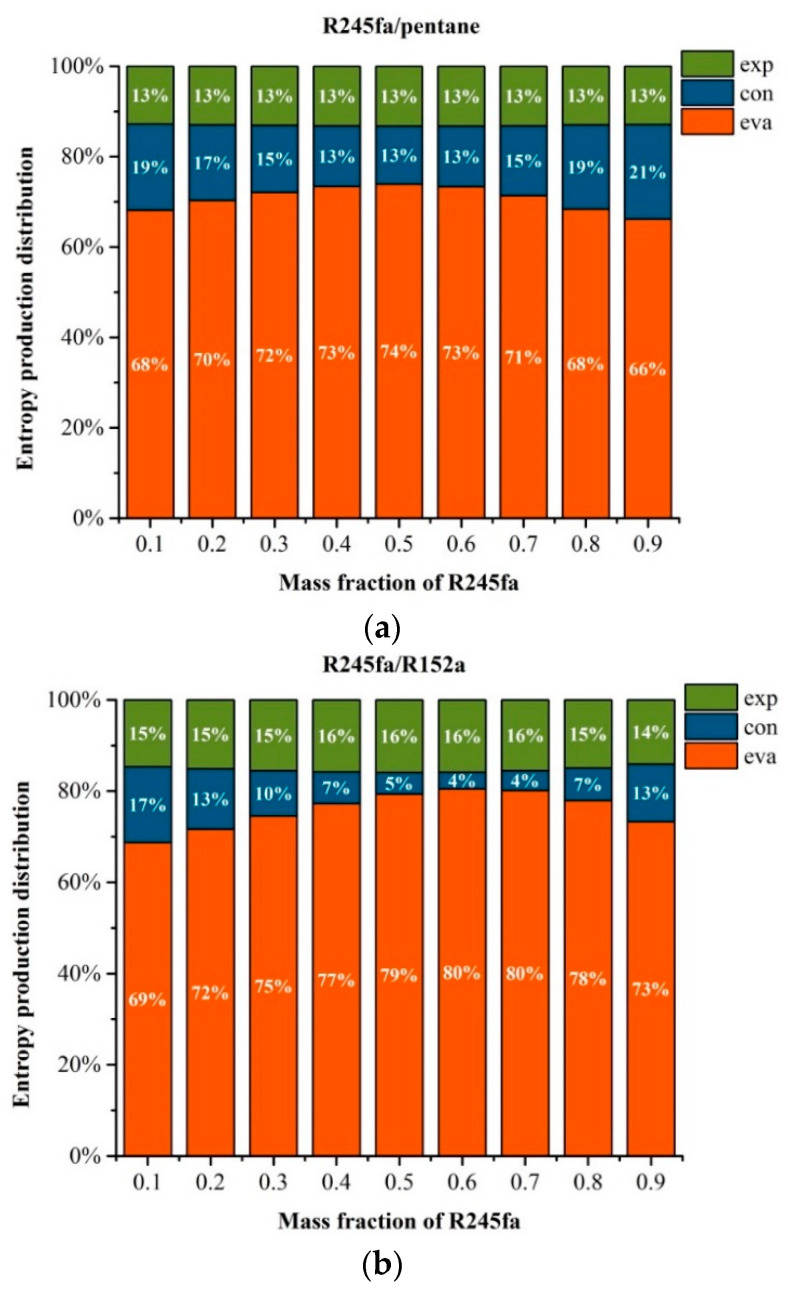

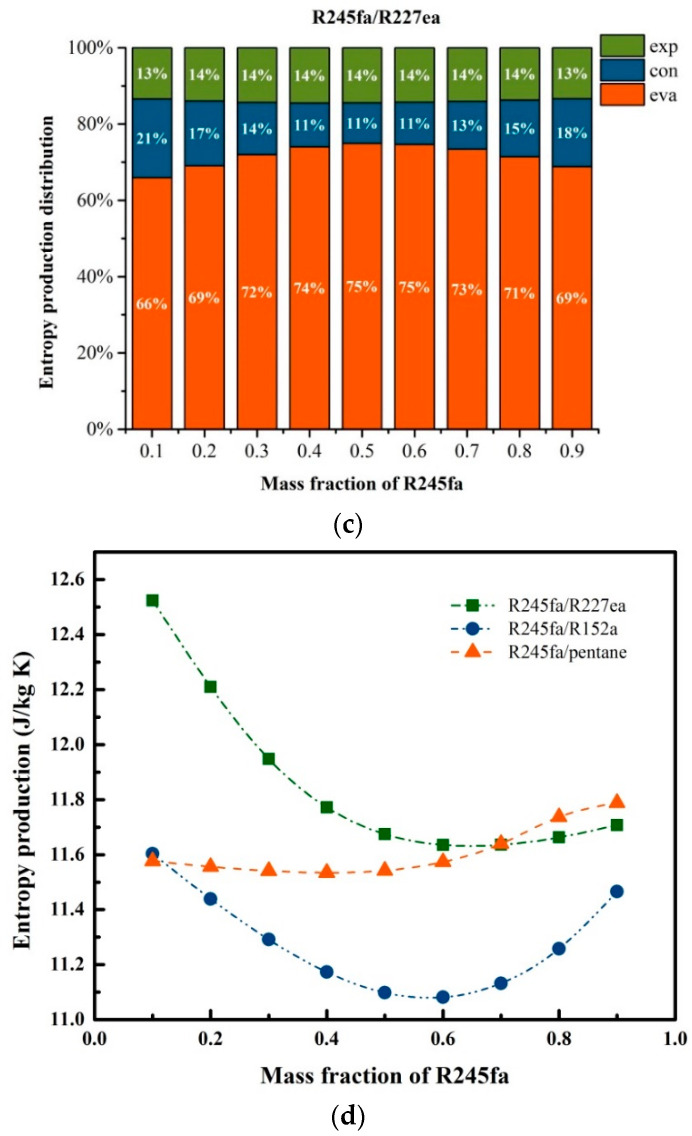
Variation of entropy generation with the mass fraction of R245fa: (**a**) R245fa/pentane; (**b**) R245fa/R152a; (**c**) R245fa/R227ea. (**d**) Variations of entropy generation with the mass fraction of R245fa using R245fa/pentane, R245fa/R152a and R245fa/R227ea.

Figure 6a–c reveals the variation of entransy dissipation with mass fraction of R245fa. The entropy generation of system is determined mainly by four parts, including *S*_g,evp_, *S_g,_*_con_, *S*_g,p_ and *S*_g,exp_. It should be noted that the *S*_g,__p_ is relatively smaller than the others. Taking R245fa/pentane as an example and for a specified mass of fraction of 0.2, *S*_g,evp_ is 8.497 J/(kg·K), *S*_g,exp_ is 2.026 J/(kg·K) and *S*_g,con_ = 1.566 J/(kg·K), while *S*_g,__p_ is 0.012 J/(kg·K). Therefore, *S*_g,__p_ can be ignored and the residual data are plotted in Figure 6. The entropy generation distributions at the expander for R245/pentane, R245fa/R152a and R245fa/R227ea are approaching 13%, 15% and 14%, respectively. Meanwhile, for the three working fluids, the entropy generation at the expander increases first and then decreases, whereas that at the condenser presents a reverse trend with the mass fraction of R245fa. The entropy generation distributions at the evaporator for R245/pentane, R245fa/R152a and R245fa/R227ea are in ranges of 66–74%, 68–80% and 66–75%, respectively, with the corresponding entropy generation distribution ranges at the condenser of 13–21%, 4–17% and 11–21%, respectively, which is shown in Figure 6a–c. Moreover, the maximum entropy generation at the expander appears for the mass fraction of 0.5 using R245, corresponding to the minimum entropy generation at the condenser. This indicates that the mass fraction has a significant influence on the entropy generation. As observed in Figure 6d, when the mass fraction of R245fa is 0.1, R245fa/R227ea owns the highest entropy generation, whereas R245f/R152a and R245fa/pentane yield a similar entropy generation. As the mass fraction of R245fa rises, the entropy generation variations for R245fa/R227ea and R245fa/R152a have a similar trend, that is, the entropy generation decreases first and then increases. Meanwhile, R245fa/pentane exhibits a slight variation with the mass fraction of R245fa. For a specified mass fraction of R245fa, R245fa/R227ea owns the highest entropy generation, whereas the lowest entropy generation is obtained by R245fa/R152a. It can also be found that the optimal mass fraction of R245fa for the minimum entropy generation is 0.6 using R245fa/R152a. 

## 6. Conclusions

In this study, the entropy and entransy dissipation analyses of a basic ORC system using three mixture working fluids (R245fa/R227ea, R245fa/R152a and R245fa/pentane) have been investigated. The effects of the four operation parameters (evaporator outlet temperature, condenser temperature, PPTD, degree of superheat), as well as the mass fraction, on entransy dissipation and entropy generation, were examined. The main results are summarized as follows:

(1) The entransy dissipations for the three mixture working fluids keep increasing with the condenser temperature, but have a nonlinear variation with the evaporator outlet temperature. The entropy generation for the three working fluids has a similar behavior of an increase with the degree of superheat and PPTD.

(2) The entransy dissipations of the R245fa/R227ea and R245fa/R152a keep rising, whereas that of R245fa/pentane yields a reverse trend with the mass fraction of R245fa. Meanwhile, the entropy generation of expander increases first and then decreases, whereas that of condenser presents a reverse trend with the mass fraction of R245fa.

(3) The entropy generation distributions at the evaporator for R245/pentane, R245fa/R152a and R245fa/R227ea are in ranges of 66–74%, 68–80% and 66–75%, respectively, with the corresponding entropy generation distribution ranges at the condenser of 13–21%, 4–17% and 11–21%, respectively.

(4) For a specified mass fraction of R245fa, R245fa/R227ea owns the highest entropy generation, whereas the lowest entropy generation is obtained by R245fa/R152a. It can also be found that the optimal mass fraction of R245fa for the minimum entropy generation is 0.6 using R245fa/R152a.

## Figures and Tables

**Table 1:** Main properties of the working fluids.

NO.	Working Fluids	M (kg·kmol^−1^)	*T*_cr_(K)	*P*_cr_(MPa)	*T*_boiling_(K)
1	R245fa	134.05	427.05	3.65	288.29
2	R227ea	170.03	374.90	2.93	256.81
3	R152a	66.051	386.41	4.51	249.13
4	Pentane	86.175	507.82	3.03	341.86

**Table 2:** Main assumptions for the ORC system.

Item	Unit	Value
Heat sources temperature	°C	120
Expander isentropic efficiency	%	80
Pump isentropic efficiency	%	80
Mass flow of heat sources	kg·s^−1^	0.5
Cooling water temperature	°C	10
Evaporator outlet temperature	°C	60
Degree of superheat	°C	10
PPDT in evaporator	°C	15
Condenser temperature	°C	20
Environmental temperature	°C	20

## Nomenclature

ORC	organic Rankine cycle
*h*	specific enthalpy, kJ/kg
*m*	mass flow rate, kg/s
*Q*	heat exchange power, kW; ratio of vapor moles to total moles
*s*	specific entropy, J/(kg·K)
*T*	temperature, K
W	power, kW
η	efficiency
*i*	state points
glide	glide temperature
cr	critical point
in	inlet
l	liquid
out	outlet
sup	degree of superheat
wf	working fluid
con	condenser
eva	evaporator
col	cooling water
exp	expander
sys	system
env	environment
h	heat source
p	pump
PPTD	pinch point temperature difference
